# Heart rate variability, a potential assessment tool for identifying anxiety, depression, and sleep disorders in elderly individuals

**DOI:** 10.3389/fpsyt.2025.1485183

**Published:** 2025-01-23

**Authors:** Wenna Liu, Shutong Wang, Hanyang Gu, Rong Li

**Affiliations:** Department of Geriatrics, Xijing Hospital, The Fourth Military Medical University, Xi'an, China

**Keywords:** anxiety, depression, sleep disorders, heart rate variability, elderly individuals

## Abstract

**Introduction:**

This study investigates how anxiety, depression, and sleep disorders impact heart rate variability (HRV) in the elderly, exploring the clinical implications of HRV changes.

**Methods:**

We examined 355 patients (163 men, 192 women) at Xijing Hospital from July 2021 to December 2022 during health check-ups. Demographics were recorded, and emotional status was assessed using the Hamilton Anxiety Scale (HAMA) and the Hamilton Depression Scale (HAMD). The Pittsburgh Sleep Quality Scale (PSQI) evaluated sleep quality. Patients were categorized into groups A-G based on the presence of emotional states and sleep disorders. HRV indices—SDNN, SDANN, RMSSD, PNN50, LF/HF, LF, and HF—were analyzed using ANOVA and multivariate logistic regression.

**Results:**

No statistically significant differences were observed in demographic, clinical, and lifestyle factors across the eight groups. Variables assessed included age, sex, body mass index (BMI), fasting blood glucose, glycated hemoglobin (HbA1c), blood lipids, blood pressure, heart rate, and histories of smoking and alcohol consumption. Additionally, the presence of hypertension, diabetes, coronary heart disease, marital status, income, and education level were evaluated, with all showing equivalence (*P* > 0.05). Significant differences in HRV indices were observed across groups, particularly in group G (patients with anxiety, depression and sleep disorders), which showed decreased HRV parameters except LF/HF, and group H (control group), which showed increased parameters, also except LF/HF (*P* < 0.01). Anxiety was an independent risk factor for reduced SDNN, SDANN, and LF (*P* ≤ 0.01), and increased LF/HF ratio (*P* < 0.01). Depression was linked to decreased SDNN, RMSSD, PNN50, and HF (*P* < 0.05). Sleep disorders independently predicted reduced PNN50 and SDANN (*P* < 0.01).

**Conclusion:**

HRV indices of individuals with varying emotional states and sleep disorders exhibited varying degrees of decrease. Anxiety, depression, and sleep disorders presented a superimposed effect on HRV. SDNN, SDANN, RMSSD, PNN50, HF and LF of HRV are of great reference value in the diagnosis of emotional and sleep disorders. For elderly patients experiencing cognitive impairment, HRV is anticipated to serve as a convenient and effective tool for assessing mood and sleep disorders.

## 1 Introduction

The aging population has emerged as a pressing global social issue, as evidenced by the data from China’s seventh population census (1). The elderly population in China is rapidly increasing, and the momentum of population growth is diminishing, with a negative growth trend becoming evident. This leads to a progressive escalation in the socio-economic and pension burden (2). The quality of life for the elderly hinges on both physical and mental health. While the elderly often prioritize physical health, societal attention must also be directed towards their mental well-being. A meta-analysis has revealed that the prevalence of depression among the elderly in our country has reached levels as high as 25.55% (3), with anxiety symptoms affecting approximately 22.11% of the older population (4). Additionally, sleep disorders have been found to affect 46.0% of elderly individuals (5). In efforts to enhance the well-being of the elderly, medical treatment should not only focus on physical ailments but also on the maintenance of their mental health.

Heart rate variability (HRV) is defined as the variance in time intervals between consecutive heartbeats. It is a collection of parameter values utilized to quantify variations in the R-R interval through various algorithms, serving as a precise indicator of alterations in the autonomic nervous system (6). Based on analytical characteristics, heart rate variability (HRV) indices can be categorized into linear and nonlinear types. Commonly utilized linear analysis methods include time-domain analysis (TDA) and frequency-domain analysis (FDA) (7). Nonlinear indices encompass the Poincare plot and sample entropy, among others. TDA, which statistically characterizes continuous heartbeat intervals, comprises the following key metrics: Standard deviation of normal-to-normal intervals (SDNN), which reflects overall HRV and serves as an indicator for evaluating the balance between sympathetic and parasympathetic nervous system activity; the standard deviation of the averages of 5-minute RR intervals (SDANN), which assesses sympathetic nerve activity; and root mean square of successive RR interval differences (RMSSD) and the percentage of successive RR intervals differing by more than 50 ms (PNN50), which predominantly reflect parasympathetic nervous system activity (8). FDA examines the distribution of heart rate time series across various frequency bands using Fourier transformation. Key indicators include low frequency (LF, 0.04-0.15 Hz), which reflects the combined activity of the sympathetic and parasympathetic nervous systems, primarily indicating sympathetic nerve activity; high frequency (HF, 0.15-0.4 Hz), which predominantly reflects parasympathetic activity via the vagus nerve; and the low frequency to high frequency ratio (LF/HF), a standard indicator for assessing the balance between sympathetic and parasympathetic systems (9). Collectively, linear HRV analysis provides a deeper understanding of cardiovascular health and autonomic nervous system function. This non-invasive tool is utilized to assess cardiac sympathetic and vagal tone, determining the equilibrium between them (10), and is extensively applied in clinical and research settings. Long-term anxiety and depression, along with associated negative emotions and sleep disorders, may result in varying levels of autonomic nervous dysfunction, thereby impacting disease progression (11). The consensus within the academic community is that HRV serves as a valuable indicator for assessing the severity and prognosis of clinical anxiety and depression (12). Nevertheless, there remains a dearth of quantitative metrics and corresponding clinical investigations regarding alterations in autonomic nervous function among elderly individuals with anxiety, depression, and sleep disorders in comparison to their healthy counterparts. Furthermore, the utility of anxiety, depression, and sleep disorder scales for evaluating these conditions in older adults with cognitive impairment is constrained. Therefore, this study aims to examine alterations in autonomic nervous function among elderly individuals with anxiety, depression, and sleep disorders through the utilization of the HRV index in a clinical setting. It is anticipated to offer clinical utility with objective, accurate and simple evaluation indicators for elderly patients in the future.

## 2 Methods

### 2.1 Subjects of study

This case-control study involved the continuous collection of information from inpatients and outpatients who underwent mental status and sleep quality evaluations at Xijing Hospital between July 2021 and December 2022. The sample size was determined based on the prevalence of anxiety, depression, and sleep disorders among elderly individuals, with exclusion criteria applied to cases that dropped out during the study or failed to meet data requirements. Ultimately, 313 patients were selected for the study group. Control group samples were primarily drawn from the inpatient and outpatient populations undergoing physical examinations at Xijing Hospital, resulting in a total of 42 participants.

The participants were randomly assessed using the Hamilton Anxiety Scale (HAMA), Hamilton Depression Scale (HAMD), and Pittsburgh Sleep Quality Scale (PSQI) scales by researchers with professional training in psychosomatic medicine to ensure alignment between the participants’ actual psychological and sleep conditions and the scale scores.

All subjects were grouped as follows. Group A: Anxiety group, 45 cases; Group B: Depression group, 42 cases; Group C: Sleep disorder group, 43 cases; Group D: Anxiety combined with depression group, 44 cases; Group E: Anxiety combined with sleep disorder group, 46 cases; Group F: Depression combined with sleep disorder group, 47 cases; Group G: Anxiety, depression and sleep disorder group, 46 cases; Group H: Control group, 42 cases.

Participants with psychological or sleep disorders must meet the following criteria: 1) Age ≥60 years; 2) Meeting the diagnostic criteria of the Chinese Classification of Mental Disorders-3 (CCMD-3) (13); 3) HAMDscore of ≥17 (14), HAMA score of ≥14 (15), or PSQI score of ≥10 (16); 4) Participants must agree to accept a 24-hour ECG monitoring; 5) Participants must have complete medical records and provide signed informed consent.

Exclusion criteria for the patients include: 1) Patients with a history of atrioventricular block, atrial fibrillation, premature atrial or ventricular arrhythmias; 2) Patients diagnosed with intellectual disability, delirium, schizophrenia, or other mental illnesses according to the CCMD-3; 3) A range of factors that may impact HRV, including hyperthyroidism, anemia, infection, trauma, and other physical ailments; 4) Patients with severe organic diseases such as acute cerebral infarction, malignant tumors, and cachexia; 5) Those who have recently undergone major catastrophic events or experienced significant stress; 6) Individuals experiencing insomnia due to physical discomfort or fatigue; 7) Patients with Parkinson’s syndrome, sleep apnea syndrome; 8) Recent use of anti-anxiety and antidepressant medications; 9) Participants who have recently used medications that impact heart rate, such as beta-blockers, thyroid hormones, and digitalis within the preceding two weeks.

The inclusion criteria for the control group were as follows: 1) Individuals aged 60 years or older; 2) Scoring below 17 on the HAMD and below 17 on the HAMA, as well as below 14 points on the PSQI; 3) Willingness to participate in a 24-hour ECG monitoring; and 4) Possession of complete medical records and signed informed consent.

The exclusion criteria for the control group include: 1) Patients with a history of atrioventricular block, atrial fibrillation, premature atrial or ventricular arrhythmias; 2) Individuals meeting the diagnostic criteria for anxiety and depression according to the CCMD-3; 3) Patients diagnosed with sleep disorders based on the PSQI and subjective reports of frequent and persistent difficulty falling asleep and/or maintaining sleep, as well as dissatisfaction with sleep as determined by clinicians; 4) Individuals with conditions known to affect HRV, such as hyperthyroidism, anemia, and other physical illnesses; 5) Patients with severe organic diseases such as acute cerebral infarction, severe infection and cachexia; 6) Recent major catastrophic events or high stress levels; 7) Parkinson’s syndrome, sleep apnea syndrome, and recent use of anti-anxiety and antidepressant medications.

### 2.2 Demographic data

Demographic data was collected using a standardized questionnaire to ensure the scientific rigor and accuracy of the study, allowing for a comprehensive review of participants’ background information and health status, including age, sex, body mass index, fasting blood glucose, glycated hemoglobin, blood lipids, blood pressure, heart rate, smoking history, drinking history, hypertension history, diabetes history, coronary heart disease history, marital status, income, and education.

### 2.3 Research methods

All the participants were evaluated their psychological state using the HAMD and the HAMA. Additionally, the PSQI was utilized to assess the sleep quality of the patients. Two trained evaluators from the psychosomatic department conducted joint examinations of the patients and independently scored them, with the average scores being recorded.

The participants underwent 24-hour monitoring using a V12 dynamic ECG recorder. ECG interference signals were eliminated through computer processing, and manual filtering was used to remove artifacts and ectopic beats. The HRV index was computed using an algorithmic system. The recorded data included four time-domain analysis indicators: SDNN, SDANN, RMSSD, PNN50, and three frequency domain analysis indicators: LF, HF, LF/HF (17, 18). Prior to the examination, participants were advised to abstain from alcohol, strong tea, coffee, and strenuous exercise.

All participants underwent assessment using the HAMA consisting of 14 items and the HAMD consisting of 17 items. A diagnosis of anxiety disorder was made if HAMA scores were equal to or greater than 14, while a diagnosis of depression disorder was made if HAMD scores were equal to or greater than 17, based on the symptoms reported by the participants over the past three months. Sleep quality was assessed using the PSQI, with scores equal to or greater than 10 indicating a sleep disorder. Higher PSQI scores were indicative of poorer sleep quality.

### 2.4 statistical analysis

Normality of the variables was assessed using the Shapiro-Wilk test, and variables conforming to a normal distribution were presented as mean ± standard deviation ( $\bar{\text{x}}$ ± s). The variables exhibiting skewed distribution were analyzed using the interquartile method. Subsequently, one-way ANOVA was employed to evaluate differences in means among multiple samples that met the criteria of normal distribution and homogeneity of variance. In cases where the assumption of homogeneity of variance was violated, the Welch test was utilized. *Post-hoc* analysis was conducted using the LSD-t test to identify significant differences between specific pairs of groups. For data that deviated from normal distribution, pairwise comparisons between groups were performed using Tamhane’s T2 test. Finally, a multivariate logistic regression analysis was conducted to examine the relationship between mental health conditions (including anxiety, depression, and sleep disorders) as independent variables and the decrease in relevant indicators of HRV as dependent variables. The analysis was performed using SPSS 26.0 software, and a two-sided test with a significance level of *P*<0.05 indicated significant differences.

## 3 Results

### 3.1 comparison of clinical baseline data

There were no significant differences in age, sex, body mass index, fasting blood glucose, glycated hemoglobin, blood lipid, blood pressure, heart rate, smoking history, drinking history, hypertension history, diabetes history, coronary heart disease history, marital status, income and education among the eight groups (*P*>0.05). See **Table 1** for details.

**Table 1:** Comparison of Clinical baseline data across seven patients groups and control group.

	Group A	Group B	Group C	Group D	Group E	Group F	Group G	Group H	*P* Value
n	45	42	43	44	46	47	46	42	
Famale [n(%)]	25(55.6)	22(47.6)	24(55.8)	27(61.4)	20(43.5)	29(61.7)	26(56.5)	19(45.2)	0.581
Age (year)	69.7 ± 6.2	69.3 ± 8.4	70.8 ± 13.0	70.7 ± 8.3	68.9 ± 8.7	72.3 ± 9.6	72.7 ± 8.7	72.0 ± 8.0	0.271
BMI (kg/m^2^)	25.2 ± 2.8	25.6 ± 3.8	24.9 ± 4.3	24.3 ± 3.2	24.7 ± 3.4	24.0 ± 2.9	23.9 ± 2.9	24.1 ± 3.0	0.147
HbA1c (%)	6.3 ± 1.6	5.9 ± 0.9	5.6 ± 1.1	6.1 ± 1.5	6.2 ± 2.2	6.5 ± 2.3	6.2 ± 1.2	6.0 ± 1.1	0.262
FBG (mmol/L)	6.3 ± 2.3	5.7 ± 1.3	6.0 ± 2.3	5.9 ± 1.9	5.4 ± 1.4	6.2 ± 2.3	6.2 ± 2.0	5.5 ± 1.3	0.095
TC (mmol/L)	4.2 ± 1.1	4.3 ± 1.1	4.0 ± 1.3	4.3 ± 1.1	4.2 ± 1.0	3.9 ± 1.0	4.1 ± 1.0	3.6 ± 1.3	0.059
TG (mmol/L)	1.7 ± 1.0	1.7 ± 1.0	1.4 ± 1.1	1.4 ± 0.9	1.6 ± 1.0	1.4 ± 0.7	1.3 ± 0.6	1.2 ± 0.6	0.053
HDL (mmol/L)	1.1 ± 0.3	1.1 ± 0.2	1.2 ± 0.5	1.2 ± 0.4	1.2 ± 0.3	1.1 ± 0.4	1.3 ± 0.4	1.2 ± 0.4	0.125
LDL (mmol/L)	2.6 ± 0.9	2.6 ± 0.9	2.3 ± 1.1	2.5 ± 0.9	2.5 ± 0.9	2.3 ± 0.8	2.3 ± 0.9	2.0 ± 1.1	0.054
SBP (mmHg)	130 ± 16	128 ± 15	130 ± 20	132 ± 19	128 ± 15	130 ± 18	135 ± 21	127 ± 16	0.425
DBP (mmHg)	77 ± 10	76 ± 11	79 ± 13	74 ± 12	78 ± 11	74 ± 14	76 ± 12	73 ± 11	0.128
HR	65.9 ± 9.6	70.3 ± 10.3	69.7 ± 8.7	67.3 ± 9.2	65.9 ± 9.1	66.2 ± 8.8	66.7 ± 7.8	65.8 ± 9.0	0.111
Smoking [n(%)]	14(31.1)	16(38.1)	13(30.2)	15(34.1)	18(39.1)	16(34.0)	17(37.0)	15(35.7)	0.973
Drinking [n(%)]	13(28.9)	7(16.7)	8(18.6)	9(20.5)	14(30.4)	14(29.8)	12(26.1)	8(19.0)	0.719
Hypertension[n(%)]	29(64.4)	25(59.5)	26(60.5)	31(70.5)	35(76.1)	38(80.9)	36(78.3)	25(59.5)	0.074
Diabetes [n(%)]	9(20.0)	10(23.8)	8(18.6)	11(25.0)	7(15.2)	15(31.9)	13(28.3)	9(21.4)	0.623
CHD[n(%)]	18(40.0)	19(45.2)	20(46.5)	22(50.0)	17(37.0)	25(53.2)	21(45.7)	19(45.2)	0.797
Average annual income (ten thousand yuan)	6.1 ± 2.2	6.5 ± 2.7	5.2 ± 2.4	5.5 ± 2.5	5.3 ± 2.5	5.4 ± 2.3	5.2 ± 2.5	6.0 ± 2.5	0.114
Married [n(%)]	43(95.6)	41(97.6)	43(100)	42(95.5)	43(93.5)	45(95.7)	45(97.8)	39(92.9)	0.733
Education years	3.7 ± 2.2	3.5 ± 2.3	3.3 ± 2.7	4.3 ± 2.8	4.2 ± 3.1	4.5 ± 2.8	4.6 ± 2.7	4.5 ± 2.8	0.135

The measurement data in the table is expressed as ( $\bar{\text{x}}$ ± s), and the number of counting data is expressed as [n (%)]. HbA1c, glycosylated haemoglobin; FBG, fasting blood glucose; TC, serum total cholesterol; TG, triglyceride; HDL, high density lipoprotein; LDL, low density lipoprotein; SBP, systolic blood pressure; DBP, diastolic blood pressure; HR, heart rate; CHD, coronary heart disease.

### 3.2 HRV analysis

HRV serves as a valuable metric for assessing autonomic nervous system function, particularly in its capacity to reflect the equilibrium between sympathetic and vagal nerve activity. It has been widely used in different areas of research, from basic research to clinical applications to assess health and disease states. Consequently, this study involved a comparative analysis of diverse HRV indices across the eight groups, with the control group exhibiting HRV indices superior to the reference values, whereas the remaining groups demonstrated a decrease in varying degrees relative to the control group. This comparison provides insights into the differential impact on autonomic function as reflected by HRV across different study groups.

Given that this study incorporated the three exposure factors of anxiety, depression, and sleep disorders, it is meaningful to make comparisons between groups with overlapping exposure factors and between groups with exposure factors and normal people. This approach allowed for a more nuanced analysis of how each factor might independently contribute to the observed outcomes. The results showed that there were significant differences in HRV parameters such as SDNN, SDANN, RMSSD, PNN50, LF/HF, LF, HF among different groups. In comparison to Group A and Group B, the HRV parameters of Group D exhibited a decrease; in comparison to Group A and Group C, the HRV parameters of Group E, with the exception of LF/HF and HF, also showed a decrease; in comparison to Group B and Group C, all HRV parameters of Group F displayed a decrease. Group G demonstrated a significant decrease in HRV parameters, excluding LF/HF, when compared to the other groups, while Group H showed an increase in HRV parameters, excluding LF/HF, with a statistically significant difference (*P*<0.01).

Individuals with sleep disorders often utilize sedatives and hypnotics, including medications like diazepam, eszopiclone, and zolpidem tartrate; thus, it is not feasible to entirely exclude participants using these drugs. Acknowledging the potential influence of sedatives and hypnotics on HRV outcomes, this study compared HRV indicators between groups that included individuals on sedatives and hypnotics (C, E, F, and G) and those who had never used such medications (C*, E*, F*, and G*). Furthermore, the trend in HRV changes was consistent across the relevant groups. Refer to **Table 2** for detailed data.

**Table 2:** Comparison of HRV among seven patients groups and control group.

Groups/Values	n	SDNN	SDANN	RMSSD	PNN50(%)	LF/HF	LF	HF
Group A	45	112.98 ± 29.22#^~△☆□	97.71 ± 22.88#^~△☆□	55.47 ± 46.18#^~△☆□	23.26 ± 23.43#^~△☆□	2.66 ± 1.23#	395.17 ± 261.16#^~△☆□	165.72 ± 109.78#~△□
Group B	42	117.79 ± 31.50#&~△◇□	109.21 ± 25.48#&~△◇□	47.62 ± 51.28#&~△◇□	14.67 ± 16.96#&~△◇□	1.11 ± 0.72#&◇	353.41 ± 158.76#&~△◇□	459.04 ± 418.56#&~△◇□
Group C	43	127.19 ± 25.25^&~△	114.19 ± 34.46^&~△	59.21 ± 45.65^&~△	33.14 ± 21.83^&~△	1.10 ± 0.52&	746.79 ± 262.27^&~△	779.50 ± 346.13&~△
Group D	44	92.75 ± 17.54~△□	81.70 ± 24.33~△□	22.30 ± 8.48~△□	5.04 ± 9.67~△□	1.11 ± 0.66	131.82 ± 90.28~△□	134.52 ± 76.75~△□
Group E	46	107.00 ± 19.08~△	97.46 ± 27.16~△	28.13 ± 13.16~△	5.07 ± 4.17~△	2.18 ± 3.13	331.30 ± 193.51~△	232.43 ± 142.03~△
Group F	47	100.09 ± 28.24~△	92.64 ± 40.02~△	41.36 ± 24.70~△	9.61 ± 7.71~△	0.65 ± 0.70	126.03 ± 62.35~△	284.85 ± 157.36~△
Group G	46	82.93 ± 29.70△	71.43 ± 26.69△	17.28 ± 6.58△	1.42 ± 1.95△	1.07 ± 0.59	73.61 ± 75.49△	80.99 ± 76.53△
Group H	42	183.38 ± 55.04~□	134.98 ± 47.81~□	198.02 ± 92.46~□	53.66 ± 26.72~□	0.61 ± 0.47	7502.09 ± 7246.60~□	16562.93 ± 16328.04~□
Group C*	11	137.45 ± 21.02☆◇□△	130.64 ± 35.34☆◇□△	89.91 ± 43.295☆◇□△	44.88 ± 19.37☆◇□△	1.03 ± 0.43◇	887.87 ± 202.81☆◇□△	959.94 ± 335.05◇□△
Group E*	11	88.45 ± 14.75 □△	71.73 ± 31.55□△	30.36 ± 19.13□△	4.32 ± 4.47□△	1.70 ± 0.80	294.76 ± 140.80□△	154.39 ± 76.75□△
Group F*	10	75.70 ± 13.26 □△	67.00 ± 13.65□△	32.20 ± 12.35□△	7.25 ± 7.71□△	0.94 ± 1.19	130.19 ± 62.92□△	292.76 ± 237.49□△
Group G*	7	48.86 ± 9.51△	38.00 ± 5.32△	15.29 ± 5.31△	1.55 ± 2.02△	1.06 ± 0.81	41.86 ± 20.17△	61.41 ± 45.54△
P value		<0.01	<0.01	<0.01	<0.01	<0.01	<0.01	<0.01
F value		42.25	16.47	73.90	51.20	13.55	44.31	44.42
P* value		<0.01	<0.01	<0.01	<0.01	<0.01	<0.01	<0.01
F* value		35.48	43.96	19.11	28.19	22.82	23.60	23.80

SDNN, standard deviation of normal to normal intervals; SDANN, the standard deviation of the averages of 5-minute RR intervals; RMSSD, root mean square of successive RR interval differences; PNN50, percentage of successive RR intervals that differ by more than 50 ms; LF, low frequency; HF, high frequency. *:Groups or values analysed among people without taking sedative or hypnotic drugs. #P<0.01 versus D; ^P<0.01 versus E; &P<0.01 versus F; ~P<0.01 versus G; △P<0.01 versus H; ☆P<0.01 versus E*; ◇P<0.01 versus F*; □P<0.01 versus G*.

### 3.3 The effect of anxiety on HRV

This study focused on a cohort of patients diagnosed with anxiety, depression, and sleep disorders to investigate the potential influence of these prevalent mood disorders on HRV. Multivariate logistic regression analysis was employed to examine the association between anxiety, depression, and sleep disorders with various indicators of HRV, including SDNN, SDANN, RMSSD, PNN50, LF, HF, and LF/HF, as dependent variables. The presence of anxiety, depression, and sleep disorders were considered as independent variables in the model. Various confounding factors, including BMI, smoking history, drinking history, hypertension, diabetes, and coronary heart disease, were controlled for in the study of patients with anxiety, depression, and sleep disorders. Regression analysis revealed a significant association between anxiety and multiple HRV indicators. Specifically, anxiety was identified as an independent risk factor for reduced SDNN, SDANN, and LF (*P ≤* 0.01). Furthermore, the analysis indicated that anxiety was also an independent risk factor for an increase in the LF/HF ratio (*P* < 0.01). Please refer to **Table 3** for further information.

**Table 3:** Logistic regression analysis was used to analyze the effect of anxiety patients on HRV.

HRV parameters	*β*	Wald χ^2^	*P*	*OR*	*95%CI*
SDNN(ms)	1.253	6.443	0.01	3.50	1.33∼9.03
SDANN(ms)	1.255	6.836	0.01	3.51	1.37∼8.99
LF/HF	-3.717	34.922	<0.01	0.24	0.01∼0.08
LF(Hz)	2.779	17.136	<0.01	16.10	4.32∼60.01

### 3.4 The effect of depression on HRV

The study investigated the impact of depression on HRV by examining indicators such as SDNN, RMSSD, PNN50, and HF as dependent variables, with anxiety, depression, and sleep disorders as independent variables. Multivariate logistic regression analysis revealed that depression was identified as an independent risk factor for the decrease in SDNN, RMSSD, PNN50, and HF (*P* < 0.05). Further details can be found in **Table 4**.

**Table 4:** Logistic regression analysis was used to analyze the effect of depression patients on HRV.

HRV parameters	*β*	Wald χ^2^	*P*	*OR*	*95%CI*
SDNN(ms)	1.19	5.63	0.02	3.28	1.23∼8.76
RMMSD(ms)	2.06	17.60	<0.01	7.81	2.99∼20.39
PNN50(ms)	3.64	31.87	<0.01	38.00	10.75∼134.36
HF(Hz)	2.33	11.99	<0.01	10.92	2.75∼38.51

### 3.5 The effect of sleep disorders on HRV

In a manner akin to the research methodologies employed in studies on anxiety and depression, the present study designated the decrease in HRV index as the dependent variable, with the presence of anxiety, depression, and sleep disorder serving as the independent variables. Following logistic regression analysis, it was determined that sleep disorder emerged as a significant independent risk factor for the reduction of PNN50 and SDANN (*P* < 0.01). Further information can be found in **Table 5**.

**Table 5:** Logistic regression analysis was used to analyze the effect of patients with sleep disorders on HRV.

HRV parameters	*β*	Wald χ^2^	*P*	*OR*	*95%CI*
PNN50(ms)	1.25	6.69	<0.01	3.48	1.35∼8.95
SDANN(ms)	1.72	12.45	<0.01	5.61	2.15∼14.61

## 4 Discussion

HRV serves as a comprehensive measure of the regulation of the cardiac autonomic nervous system, encompassing frequency domain, time domain, and nonlinear indices. Frequency domain indicators such as LF、HF、LF/HF, time domain indicators such as SDNN、SDANN、RMSSD、PNN50, and non-linear indicators such as Poincare map and sample entropy are utilized to assess the complexity and non-linear characteristics of HRV. It is widely accepted among scholars that SDNN primarily signifies the overall activity of the autonomic nerve system. SDANN and LF are indicative of sympathetic nerve activity, with their values decreasing as sympathetic nerve tension increases. RMSSD、PNN50 and HF, on the other hand, reflects vagal nerve activity, with its value decreasing as vagal nerve tension reduces. The LF/HF ratio serves as a quantitative measure for assessing the functional equilibrium of the sympathetic and vagus nerves. The interpretation of HRV indices remains inconclusive. A study suggests that while LF is modulated by sympathetic nerve function, it is not advisable to rely solely on LF to gauge sympathetic nervous system activity, as it is also impacted significantly by vagus nerve activity and other factors. LF/HF can offer insights into the regulatory function of the autonomic nervous system, however, its accuracy is compromised by nonlinear relationships and various influencing factors (19).

In this research, the elderly population was categorized into groups based on varying emotional states and sleep patterns to analyze discrepancies in HRV indicators among the eight groups. Findings revealed that individuals combined with anxiety, depression, and sleep disorders exhibited a decrease in all HRV metrics, with the exception of LF/HF, compared to the normal control group, with the most pronounced decrease observed in this cohort. Specifically, individuals with comorbid mood and sleep disturbances experienced a more substantial decline in HRV compared to those with singular anxiety, depression, or sleep disorders. This suggests that anxiety, depression, and sleep disorders may contribute to a reduction in HRV and exhibit a synergistic effect, a phenomenon not previously explored in existing literature. Consequently, our findings propose a novel hypothesis: a pronounced decrease in HRV may correlate with heightened emotional instability in patients and poorer sleep quality (20, 21).

Recent research has increasingly demonstrated the impact of mood disorders, such as anxiety and depression, on HRV (22). In particular, depressed mood has been associated with a heightened susceptibility to various cardiac ailments (23). While existing research has examined the correlation between depression and HRV (24), there remains a dearth of studies focusing on this relationship within the elderly demographic. This investigation utilized multivariate logistic regression analysis to investigate the distinct influence of anxiety and depression on HRV among elderly individuals. The findings revealed that anxiety independently posed a risk for the diminishment of SDNN, SDANN, and LF (*P*<0.05). This suggests that individuals with anxiety may experience a decrease in HRV measures. The overview of HRV indicators and standards indicates that SDNN is influenced by both the sympathetic nervous system (SNS) and the parasympathetic nervous system (PNS), reflecting their balance. SDNN measurements are highly correlated with frequency domain indicators, particularly when the low frequency (LF) power exceeds high frequency (HF) power, suggesting a greater influence of LF on SDNN (7). This study’s findings reveal that anxiety is a risk factor for reductions in both SDNN and LF. Anxiety leads to varying degrees of decrease in SDNN and LF, thereby confirming their correlation within the frequency domain index. Currently, the physiological basis of the LF band is not well understood, although many scholars believe it reflects cardiac sympathetic nerve activity (25). Nonetheless, evidence suggests that LF measurements result from both parasympathetic and sympathetic nerve activities, not solely the sympathetic activity (26, 27). This study identified anxiety as a risk factor for LF reduction, potentially due to sympathetic dominance in LF measurements when both systems are active. Given the incomplete understanding of LF’s complex effects on SDNN and its physiological implications, SDANN is recommended as a characteristic index for measuring sympathetic nerve activity, with a focus on SDANN decreases when assessing anxiety. Furthermore, the analysis indicated that anxiety is an independent risk factor for an increased LF/HF ratio. The LF/HF ratio serves as a common marker of cardiovascular health, with elevated levels potentially indicating autonomic nervous system imbalance. According to current theory, which posits that LF results from combined parasympathetic and sympathetic activity, and HF from parasympathetic activity, an elevated LF/HF ratio can be interpreted as sympathetic dominance in individuals with imbalanced autonomic nervous systems. Similarly, a depressive mood was identified as an independent risk factor for reduced SDNN, RMSSD, PNN50, and HF, implying that the mood’s impact on autonomic balance may decrease these indicators in individuals with depression. Studies have demonstrated that, compared to the general population, measurements in depressed patients, including LF, HF, LF/HF, SDNN, and RMSSD, are decreased (28). This study aligns with previous findings but underscores the clinical relevance of certain HRV indicators in reflecting depressive mood. The study’s results indicate that both anxiety and depression are risk factors for SDNN decline, with anxiety often associated with sympathetic hyperactivity and depression with parasympathetic over-inhibition, thereby confirming the dual influence of both nerves on SDNN. Currently, there is a general consensus that RMSSD is the primary time-domain measure for quantifying vagal nerve activity (22). This study corroborated these findings and suggested that PNN50 and HF are also vital indicators for assessing depressive mood, advocating for a comprehensive analysis of multiple HRV indicators when evaluating depressive mood.

To sum up, the assessment of emotional disorders in the elderly population is significantly influenced by subjective factors inherent in the scales used (29), and various limitations, such as sensory impairments and cognitive dysfunction, may hinder the accurate measurement of these scales (30). Therefore, the utilization of HRV as an objective and easily accessible clinical monitoring indicator becomes imperative. By monitoring changes in SDNN, LF, and HF, healthcare professionals can effectively identify the presence and type of mood disorder in elderly patients.

Previous research has predominantly focused on examining the impact of various physiological and psychological factors on HRV. However, the prevalence of sleep disorders among the elderly poses a significant challenge to their overall physical and mental well-being, yet the potential influence of sleep disorders on HRV in this population remains largely unexplored. It is important to note that not only physical ailments, but also mental health conditions such as anxiety and depression, can contribute to the development of sleep disorders (31). Hence, this study employed multivariate Logistic regression analysis to examine the potential influence of sleep disorders on HRV among elderly individuals. The results of the analysis indicated that sleep disorder emerged as a significant independent risk factor for the decline in PNN50 and SDANN. These findings align with some of the prior research, such as the study conducted by Trinder et al., which demonstrated a reduction in certain HRV measures, including RMSSD and PNN50, during nighttime in individuals with sleep disorders (32). Moreover, Tobaldini et al. discovered a negative correlation between sleep disturbances and HRV in a cohort of young adults. Their findings indicated that individuals with insomnia exhibited notably decreased nighttime HRV, particularly in relation to parasympathetic parameters (33). Utilizing a distinct measurement approach, another study employed polysomnography to ascertain the subject’s state of wakefulness and concurrently assessed HRV alterations. The study revealed that high-frequency (HF) components of HRV were reduced during wakefulness, with physiological variations in EEG arousal levels influencing cardiac autonomic nervous activity in healthy individuals (34). This indicates that sleep disorders may result in dysfunction of specific autonomic nervous system functions, particularly those pertaining to cardiovascular well-being. While the PSQI asks patients to report their sleep patterns over the past month, it provides an assessment of their established sleep patterns. Furthermore, this study employed a 24-hour long-term HRV recording, offering greater predictive power than a 5-minute short-term assessment. A variety of sleep quality assessment instruments have validated that sleep disorders are associated with reduced HRV. Currently, the PSQI, widely utilized, primarily depends on subjective patient evaluations, whereas HRV studies focus on analyzing the patterns and variations in human heart rate, which are not influenced by subjective biases. These results are considered more objective and reliable, and a comprehensive assessment can integrate HRV analysis when using the PSQI to evaluate sleep disorders.

The limitation of this study is the lack of a clearly defined normal threshold for various HRV parameters in healthy individuals, despite the observed decrease in these parameters in individuals with anxiety, depression, and sleep disorders, particularly when all three conditions are present simultaneously. Future extensive clinical investigations are anticipated to establish a threshold level for HRV in the elderly demographic, thereby positioning HRV as a primary objective measure for assessing emotions.

Overall, HRV is anticipated to serve as a non-invasive and readily accessible assessment tool for physicians to identify patients potentially affected by negative mood and sleep disorders (35), especially applicable to old patients with cognition impairment. Investigating the correlation between anxiety, depression, sleep disorders, and HRV among individuals will enhance comprehension of the psychological mechanisms underpinning associated illnesses and facilitate the formulation of potentially efficacious treatment strategies.

## 5 Conclusion

In the diagnosis of emotional disorders, time-domain metrics such as SDNN, SDANN, RMSSD, and PNN50, alongside frequency-domain metrics like HF and LF, hold significant reference value. For diagnosing sleep disorders, the time-domain indicators SDANN and PNN50 of HRV are particularly valuable. Among the elderly with cognitive dysfunction, the application of traditional scales is limited; however, the reduction in related indicators of HRV shows promising clinical potential for aiding in the diagnosis of anxiety, depression, and sleep disorders.

## Data Availability

The original contributions presented in the study are included in the article/supplementary material. Further inquiries can be directed to the corresponding author.
